# An open-access dashboard to interrogate the genetic diversity of *Mycobacterium tuberculosis* clinical isolates

**DOI:** 10.1038/s41598-024-75818-y

**Published:** 2024-10-21

**Authors:** Jody Phelan, Klaas Van den Heede, Serge Masyn, Rudi Verbeeck, Taane G. Clark, Dirk A. Lamprecht, Anil Koul, Richard J. Wall

**Affiliations:** 1 Department of Infection Biology, Faculty of Infectious and Tropical Disease, London School of Hygiene and Tropical Medicine, London, WC1E 7HT UK; 2 Janssen Global Public Health R&D, LLC, Janssen Pharmaceutica NV, Turnhoutseweg 30, 2340 Beerse, Antwerpen, Belgium

**Keywords:** *Mycobacterium tuberculosis*, Clinical isolate, Genetic diversity, Non-synonymous changes, Conservation, Genome informatics, Genetic databases, Genetic variation, Predictive markers, Antimicrobial resistance

## Abstract

Tuberculosis (TB) remains one of the leading infectious disease killers in the world. The ongoing development of novel anti-TB medications has yielded potent compounds that often target single sites with well-defined mechanisms of action. However, despite the identification of resistance-associated mutations through target deconvolution studies, comparing these findings with the diverse *Mycobacterium tuberculosis* populations observed in clinical settings is often challenging. To address this gap, we constructed an open-access database encompassing genetic variations from > 50,000 clinical isolates, spanning the entirety of the *M. tuberculosis* protein-encoding genome. This resource offers a valuable tool for investigating the prevalence of target-based resistance mutations in any drug target within clinical contexts. To demonstrate the practical application of this dataset in drug discovery, we focused on drug targets currently undergoing phase II clinical trials. By juxtaposing genetic variations of these targets with resistance mutations derived from laboratory-adapted strains, we identified multiple positions across three targets harbouring resistance-associated mutations already present in clinical isolates. Furthermore, our analysis revealed a discernible correlation between genetic diversity within each protein and their predicted essentiality. This meta-analysis, openly accessible via a dedicated dashboard, enables comprehensive exploration of genetic diversity pertaining to any drug target or resistance determinant in *M. tuberculosis*.

## Introduction

Tuberculosis (TB) is caused by infection with the bacterial pathogen *Mycobacterium tuberculosis* and, after COVID-19, stands as the leading infectious disease killer globally, with an estimated 1.3 million deaths in 2022^1^. The success of existing treatment regimens is challenged by their long duration and associated drug toxicity, leading to reduced compliance and the emergence of drug resistance, with approximately 0.4 million cases of drug-resistant TB infection reported in 2022^1^. To combat the threat of the increasing TB disease burden, drug discovery efforts are focused on developing shorter treatment regimens, improving the treatment of drug-resistant TB infections, and minimising drug resistance^2^.

Many new antitubercular drugs are being developed by target-based structure-enabled drug discovery^2^. However, the single-target nature of these compounds renders them more susceptible to the emergence of drug resistance through, for instance, target-based mutations such as single nucleotide polymorphisms. Identification of resistance-conferring genetic changes, such as mutations, is often the first step in target identification studies and subsequent early-stage drug development. However, these mutations and/or genetic changes are rarely compared against genetic data from clinical isolates to investigate whether resistance is already present or bacteria with these mutations are viable in the clinic. This comparison would require determining a baseline of genetic variance of every *M. tuberculosis* protein using sequencing data from clinical TB strains. This would allow investigation of whether populations with these known resistance-conferring mutations are present and thus could survive and proliferate under future drug pressure.

Previous literature has demonstrated that essential genes tend to exhibit higher levels of genetic conservation compared to non-essential genes, suggesting an evolutionary advantage of maintaining sequence integrity. Evolutionary studies across various organisms, including bacteria like *Escherichia coli* and *Bacillus subtilis*, as well as eukaryotes, have consistently observed this trend^3,4^. The conservation of essential genes across divergent species underscores their fundamental role in cellular processes and highlights their significance in maintaining fitness^5^. Additional studies have further supported these findings through evolutionary conservation analysis between essential and non-essential genes in bacterial genomes, providing additional evidence for the selective pressure acting on essential genes to maintain their sequence conservation^6,7^. Understanding the evolutionary dynamics of essential genes provides valuable insights into the genetic determinants of cellular viability and may inform strategies for drug target prioritisation and antimicrobial drug development.

Here, we established an open-access database comprising genetic variations extracted from > 50,000 clinical isolates of *M. tuberculosis*, encompassing the entire protein-encoding genome. Through comprehensive analysis, we juxtaposed the genetic diversity of each protein against sequence conservation within the *Mycobacterium* genus and gene vulnerability scores^8^. Notably, this revealed a robust correlation between essentiality and genetic conservation across the proteome. To illustrate the utility of this dataset in terms of drug discovery, we focused on four targets of compounds currently in phase II clinical development. By comparing the genetic variability of these targets with known resistance-associated mutations derived from lab-adapted strains, we pinpointed 14 amino acid positions across three drug targets containing resistance-conferring mutations observed in clinical isolates. Accessible through a dedicated dashboard, this freely available meta-analysis presents an invaluable resource for interrogating the genetic diversity of drug targets and resistance determinants within *M. tuberculosis*.

## Results

### Construction of a genetic diversity dataset from *M. tuberculosis* clinical isolates

**Fig. 1:**
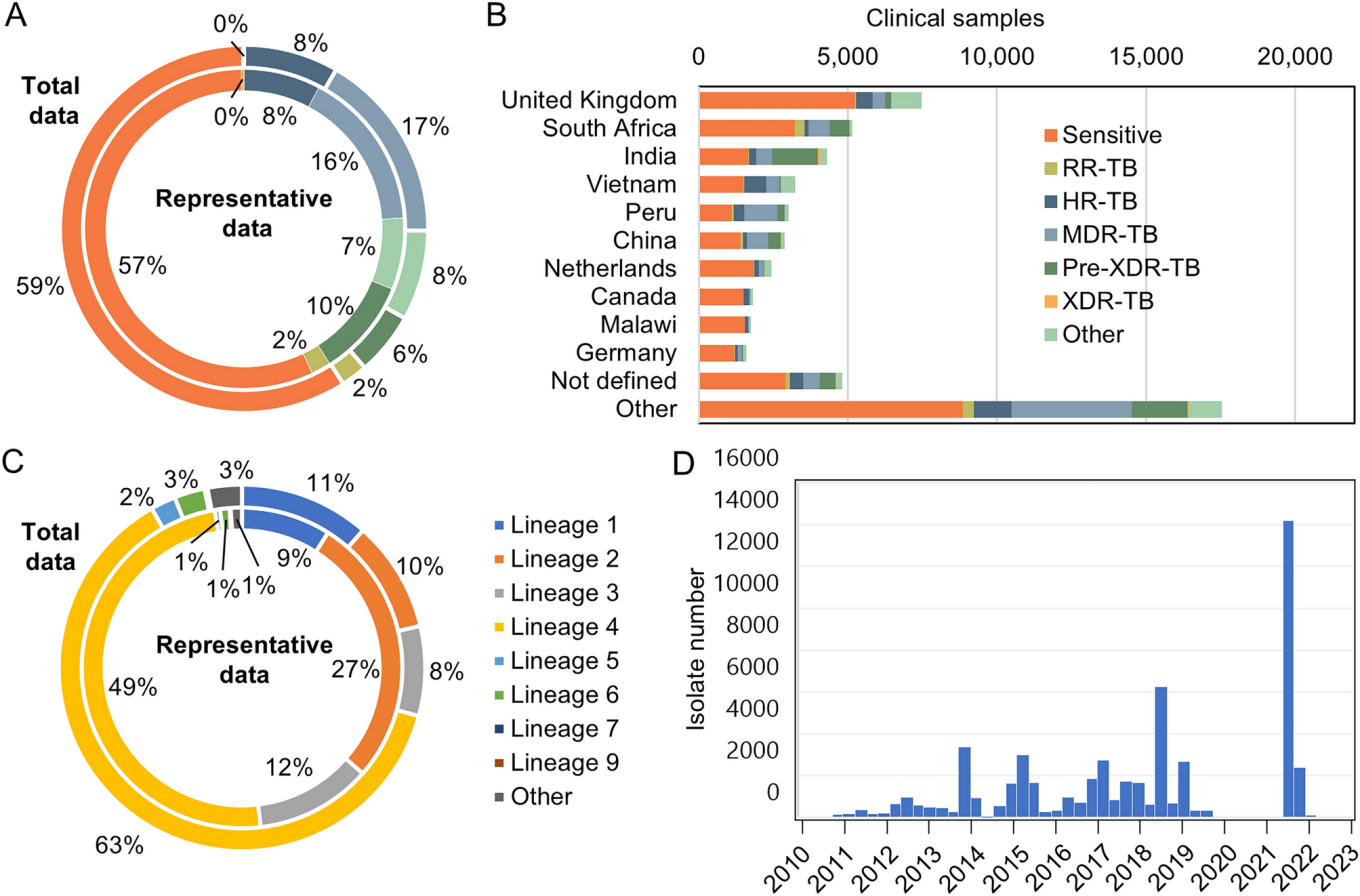
Overview of the data collection and analysis–Comparison of actual or predicted drug responsiveness for the (**A**) total dataset (outer ring) compared to the representative sample set (inner ring) and (**B**) based on the originating country for the total dataset. (**C**) Lineage of the total dataset (outer ring) compared with the representative dataset (inner ring). (**D**) Histogram of date when clinical isolate was deposited.

To compile a robust dataset for comprehensive exploration of genetic diversity, we aggregated previously deposited whole-genome sequences from clinical isolates of *M. tuberculosis* and consolidated them into an accessible dashboard. This dataset encompasses 51,183 genomic sequences obtained from TB clinical isolates derived from infected patients. Our subsequent analysis prioritised non-synonymous mutations, indels, and genomic deletions, facilitating an in-depth meta-analysis of genetic variations across every protein encoded by *M. tuberculosis*. We focused only on protein-coding genes since the intended application of this dataset was to allow investigation of the presence of target-based non-synonymous changes in circulating clinical isolates of *M. tuberculosis*. This means that genetic differences in ribosomal RNA genes including rrs and rrl, linked to resistance to streptomycin and linezolid, respectively, are not included in this analysis. A total of 1,063,811 non-synonymous changes across 694,579 sites were observed in 4029 protein coding genes. We identified an average of 173 changes per gene, and when normalised to protein length, implicated an average of 49% of sites containing a polymorphism. The conservation at sites was generally quite high, with mean gene conservation values of 99.93%. We also isolated a smaller representative dataset of 5844 samples that better reflected the underlying genetic diversity. Compared to this representative dataset, our full dataset had similar distribution of drug susceptibility (Fig. 1A). As expected, for the full dataset, samples were mainly deposited by countries with lower TB incidence but higher whole genome sequencing capacity (Fig. 1B). Indeed, most of underrepresentation was in high burden countries in the global south especially Asia (China, the Philippines, India, Indonesia). Our full dataset was slightly over-represented for lineage 4 samples and underrepresented for lineage 2 as well as lineages 5–9 (Fig. 1C). Whilst accurate metadata of the date of collection was not available for most of the clinical samples, all the sample data collated in our dataset was deposited between 2010 and 2023 (Fig. 1D). This final dataset represents a comprehensive catalogue of genetic variance from clinical isolates for every protein in *M. tuberculosis*.

To make this extensive resource more accessible to the TB drug discovery research community, we established a user-friendly interface for data interrogation (https://www.lshtm.ac.uk/research/centres-projects-groups/satellite-centre-for-global-health-discovery#genetic-diversity). This dashboard can be used to investigate the genetic variance of any protein of interest in *M. tuberculosis* whilst also providing metrics such as conservation between closely related *Mycobacterium* species.

### Genetic diversity and species conservation of genes correlate with gene vulnerability

**Fig. 2:**
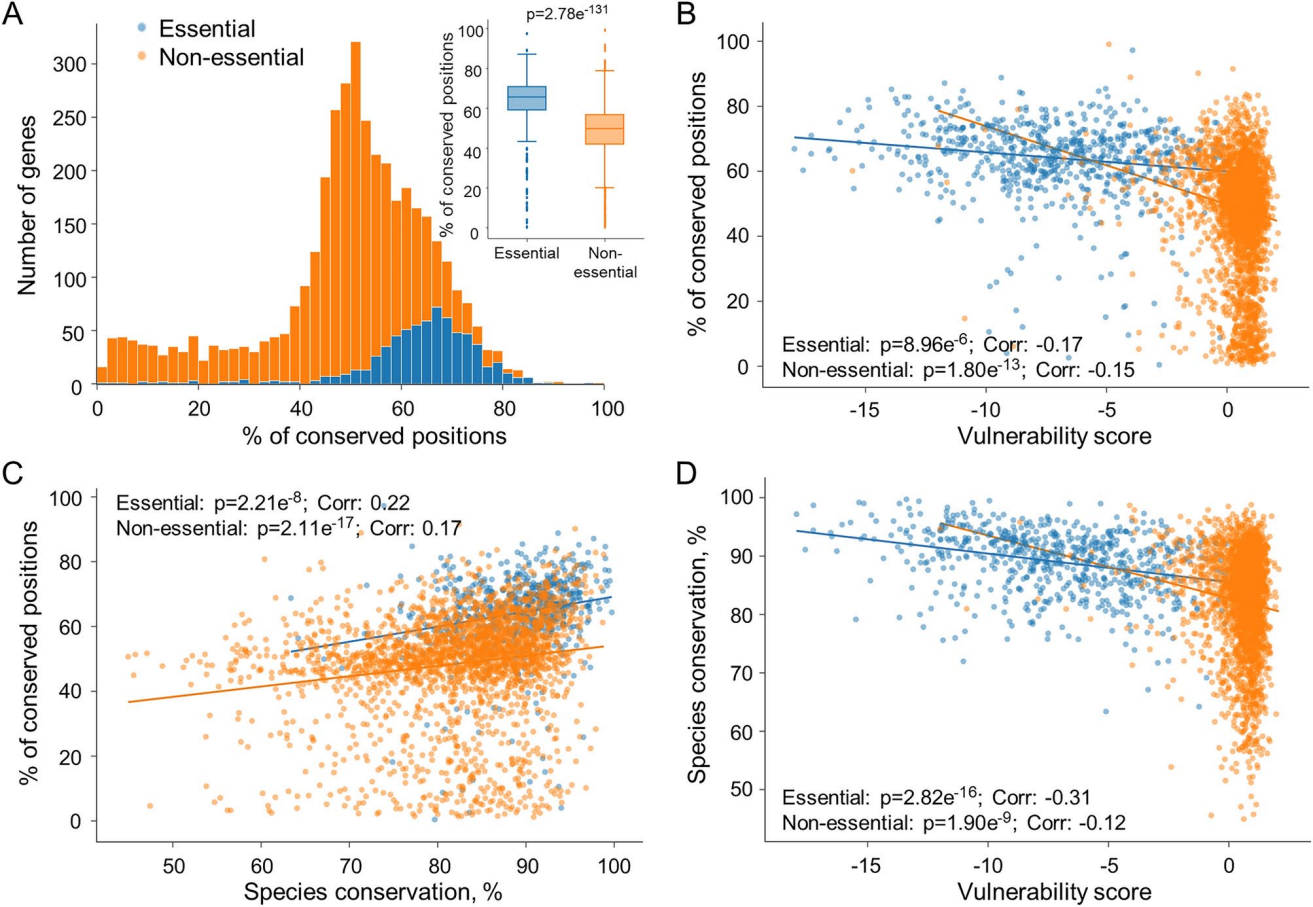
Genome-wide comparison of gene vulnerability, genetic diversity and species conservation–(**A**) Histogram of genes per percentage of polymorphic positions. Insert shows direct comparison between genes predicted to be essential verses non-essential. (**B**) Comparison of genetic diversity (% of positions that are completely conserved amongst all isolates) and gene vulnerability score^8^. (**C**) Comparison of genetic diversity and species conservation (between *mycobacterium* species). (**D**) Comparison of gene vulnerability score and species conservation. Genes are colour-coded based on essentiality.

To compare essentiality and conservation on a global scale, we used our extensive database to compare genetic variance between clinical isolates against gene vulnerability scores, a measure of gene essentiality, identified through a genome-wide CRISPRi-mediated essentiality screen^8^ (Fig. 2A,B). These relationships were investigated separately for genes classed as essential and non-essential (based on the CRISPRi-mediated essentiality screen^8^) as these groups formed distinct clusters within the feature space. Firstly, this showed a statistical difference in the level of genetic variance of coding regions between genes classed as either essential or non-essential genes (Fig. 2A). Secondly, essential genes were more likely to have a higher number of conserved positions compared to non-essential genes. Indeed, there was a clear correlation between the vulnerability score, which uses Bayesian modelling to quantify the vulnerability of each gene, and the genetic variance between clinical isolates (Fig. 2B).

Aside from genetic variance within a species, there is also evidence that genetic conservation between bacterial species is directly linked to gene essentiality^4^. The genus of *Mycobacterium* contains over 190 species, with the most commonly known members including *M. tuberculosis* and *M. leprae*, the causative agent of leprosy. Members of the genus are identified by their waxy, lipid rich cell walls consisting of mycolic acid. Within our dashboard, we have included the amino acid sequences of six species of this genus including *M. abscessus*, *M. marinum* and *Mycolicibacterium smegmatis*, together containing more than 24,000 protein sequences. Species conservation was then scored based on the average sequence identity between *M. tuberculosis* and the other species. As with essentiality and vulnerability, there was a statistically significant association between the genetic variance among clinical isolates and the species conservation when analysed via linear ordinary least squares regression (Fig. 2C). This supports previous work that suggests essential genes are more conserved between species in bacteria than non-essential genes. Finally, comparison of the species conservation and the vulnerability scores also revealed a statistically relevant correlation (Fig. 2D; Supplemental Table S1). Taken together this data suggests there is a relationship between the genetic conservation, both in clinical isolates and different species, and the vulnerability and essentiality of *M. tuberculosis* proteins (Supplemental Table S1).

### Identifying inherent drug resistance in new antitubercular drug targets

The main application of this genetic diversity dataset is to provide a baseline for genetic variance of any drug target of new or future clinical compounds to measure future population dynamics. This would identify both inherent resistance within the population but also provide an indication of whether target-based mutations observed in lab-generated resistance strains could be viable in clinical isolates. As demonstration of the utility of our dashboard, we focused on the respective drug targets of four compounds undergoing stage II clinical trials for the treatment of TB: (i) SQ109, an 1,2-ethylenediamine, that targets the mycolic acid transporter, MmpL3^9^; (ii) GSK070, an oxaborole derivative that inhibits leucyl tRNA synthetase (LeuS)^10^; (iii) BTZ-043, a benzothiazinone, shown to inhibit DprE1^11^ and; (iv) Q203 (Telacebec) an imidazopyridine amide, known to target the cytochrome *bc*_1_ complex, specifically QcrB^12^.

**Fig. 3:**
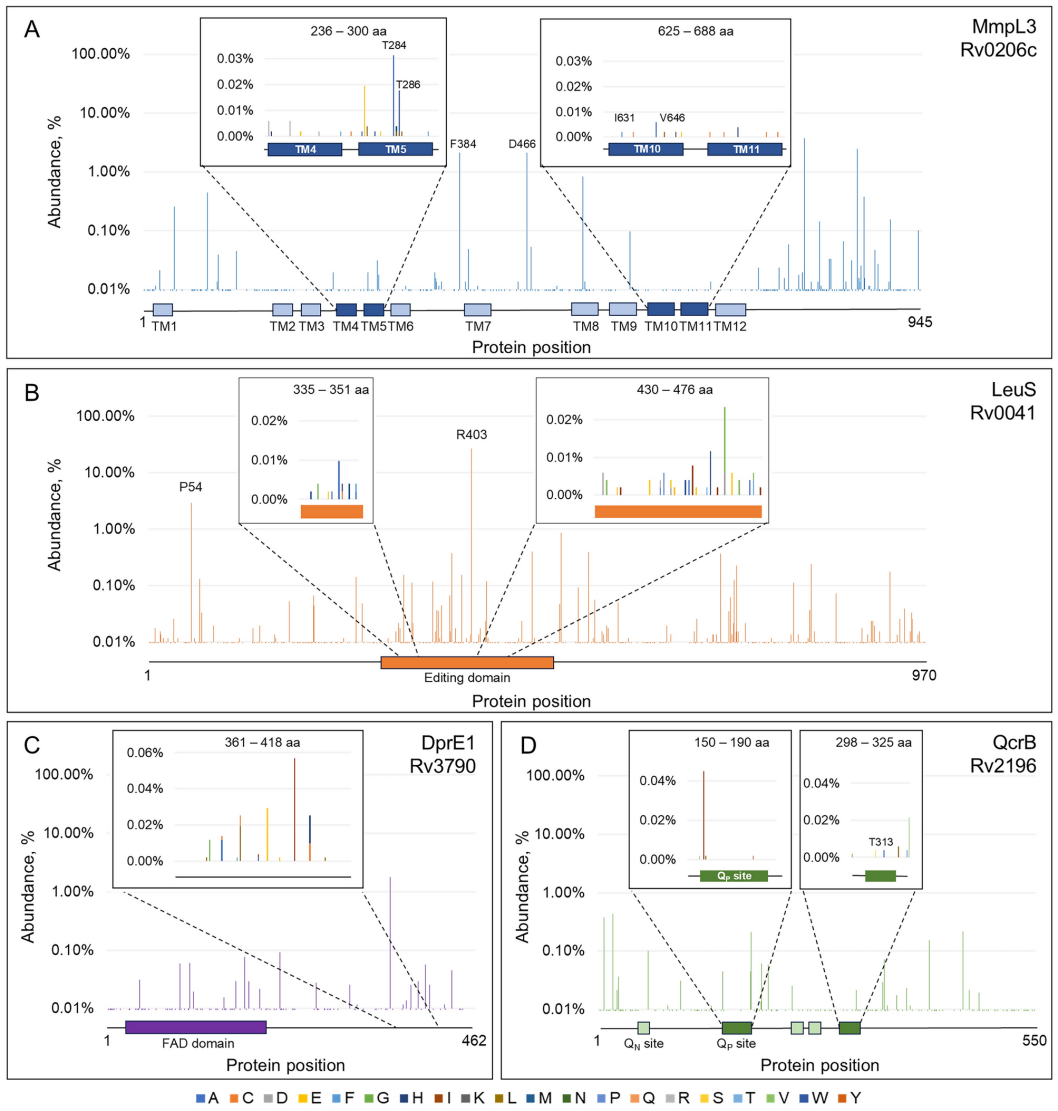
Genetic diversity of next generation drug targets in current development–Gene-wide genetic variation in (**A**) MmpL3, (**B**) LeuS, (**C**) DprE1 and (**D**) QcrB. See Supplemental Figs. S1 and S2 for sequence alignment and predicted drug binding regions for LeuS and DprE1. Inserts include regions predicted to interact with inhibitors and where mutations have been identified from lab-adapted resistance strains.

MmpL3 (Rv0206c) belongs to the Resistance, Nodulation and Division (RND) superfamily and transports trehalose monomycolate for cell wall biogenesis. While SQ109 is the most advanced compound to target MmpL3, there are multiple classes of compounds shown to inhibit this promiscuous drug target. During the development of these compounds, 136 unique amino acid changes have been identified in 83 different positions within the MmpL3 protein from a range of *Mycobacterium* species, predominately *M. tuberculosis*^13^. While many of these mutations have not been associated directly with SQ109 resistance, it is conceivable that several will lead to cross-resistance. We analysed our genetic diversity dataset to identify mutations in the MmpL3 coding sequence (Fig. 3A). SQ109 is predicted to interact with transmembrane domains (TMs) 4–5 and 10–11 (236–300 and 625–688 aa) of MmpL3, however mutations have been identified covering the whole protein^13^. Two mutations, unconnected to drug resistance, F384I and D466E, were prominent in our dataset and further investigation revealed that these mutations occurred almost exclusively in samples from lineage 6 and animal associated lineages. This suggests the mutations originated from single acquisition events and likely evolved under neutral evolution. We next compared the genetic diversity of MmpL3 with in vitro lab-generated mutations known to provide resistance to inhibitors of this target^13^. Our analysis identified genetic variance at 10 amino acid positions that can also maintain resistance-conferring mutations (Table 1; Fig. 3A). Two amino acid positions were particularly enriched with T284A occurring in 17 isolates from L4.5 originating mostly in China and Vietnam and T286M occurring in 8 isolates from L3 mostly with unknown origin as well as two isolates from the United Kingdom. In the absence of selective pressure, this would suggest that these mutations have minimal impact on bacterial growth and could be selected under drug pressure.

**Table 1:** Genetic diversity of clinical isolates compared with known resistance-conferring mutations.

Target	Gene ID	Mtb position	Mutation observed	Genetic diversity	Location of clinical isolates
Mmpl3	Rv0206c	Q40	Q40R, Q40H	Q 99.98%; R < 0.01%; − 0.02%	Portugal
		T284	T284A	T 99.95%; A 0.03%; − 0.02%	China, Vietnam
		T286	T286K (+ L320P *M. bovis*)	T 99.97%; K < 0.01%; M 0.02%; − 0.01%	UK, Thailand
		N365	N365S (+ V581A, V681I)	N 99.99%; K < 0.01%; S < 0.01%; − < 0.01%	India
		M393	M384I (*M. abscessus*)	M 99.99%; I < 0.01%; − < 0.01%	India
		M492	M492T (+ V564A, V681I)	M 99.99%; T < 0.01%; −0.01%	Malawi
		I631	I616F (+M313I *M. abscessus*)	I > 99.99%; F < 0.01%; − < 0.01%	
		V646	V646M (+ F255L)	V 99.99%; M < 0.01%; − < 0.01%	UK
		A700	A700T	A 99.99%; T < 0.01%; −0.01%	Pakistan
		V713	V713M	V > 99.99%; L < 0.01%; M < 0.01%	Ethiopia, Netherlands
LeuS	Rv0041	V482	V468L (*M. abscessus*)	V 99.98%; L 0.01%; −0.01%	Belgium, Germany, Malawi
		K516	K502E (*M. abscessus*)	K 99.99%; E < 0.01%; −0.01%	India
QcrB	Rv2196	T313	T313A	T 99.99%; A < 0.01%; − < 0.01%	
		M342	M342V	M 99.98%; V 0.01%; −0.01%	Ghana, Italy, UK

A: alanine; C: cysteine; D: aspartic acid; E: glutamic acid; F: phenylalanine; G: glycine; H: histidine; I: isoleucine; K: lysine; L: leucine; M: methionine; N: asparagine; P: proline; Q: glutamine; R: arginine; S: serine; T: threonine; V: valine; W: tryptophan; Y: tyrosine.

The oxaborole derivative, GSK070, is the most advanced of several chemical series targeting LeuS (Rv0041). The compounds are predicted to bind within the editing domain and multiple target-based mutations have been identified in in vitro experiments, predominately in the related pathogen, *M. abscessus*^14–20^ (Fig. 3B; Supplemental Fig. S1; Supplemental Table S2). The most prominent genetic variations, not connected to drug resistance, was seen at positions P54 (L4.2 associated) and R403 (L2.2 associated). These mutations occurred mostly in a single clade and thus probably only originated in the absence of selective pressure. In terms of drug resistance, two mutations–V468L and K502E (equivalent to V482 and K516 in *M. tuberculosis*)—identified in lab-adapted *M. abscessus* resistant strains were observed in our genetic diversity dataset (Table 1). DprE1 (Rv3790) is also a promiscuous target in TB drug discovery with multiple compounds inhibiting this target including BTZ-043. Indeed, a myriad of mutations providing resistance to DprE1 inhibitors have been identified from lab-adapted resistance strains largely associated with the compound binding region^11,21–26^ (Supplemental Fig. S2; Supplemental Table S3). A significant enrichment of A356T was observed which was associated with isolates from L1.2.1.2 (Fig. 3C), however, no genetic variance was observed that correlated with known resistance-conferring mutations. Finally, the cytochrome *bc*_1_ complex, specifically the QcrB subunit (Rv2196), is the target of multiple compounds including Q203 and several resistance-conferring mutations have been identified in the Q_p_ (or Q_o_) site where the compounds are predicted to bind^12,27–36^ (Supplemental Table S4). Whilst, there was no major enrichment of genetic variance in this target, two known resistance-conferring mutations—T313A and M342V–were observed (Table 1; Fig. 3D; Supplemental Table S4). It is noteworthy to mention that for all of the drug targets discussed here, the genetic variance was noticeably lower in the predicted drug binding sites compared to the surrounding protein sequence.

## Conclusion

In this study, we curated an extensive repository of genetic variants derived from whole genome sequencing data obtained from > 50,000 clinical isolates of *M. tuberculosis* collected since 2010. This resource encompasses genetic diversity profiles for more than 4000 genes of *M. tuberculosis*, conveniently accessible to the research community through a dedicated dashboard. Our analysis of this dataset unveiled a notable pattern: essential genes in Mycobacteria exhibit higher levels of conservation compared to non-essential genes, evident both within clinical isolates and across closely related species. This dataset serves as a valuable tool for exploring the genetic landscape of potential drug targets or resistance determinants. To demonstrate its utility, we focused on several targets of compounds currently undergoing phase II clinical trials. Our investigation revealed that while significant resistance was not prevalent in clinical settings, instances of laboratory-induced resistance-conferring mutations were detected in clinical isolates. Importantly, our findings suggest that these mutations do not compromise bacterial survival in patients and could therefore potentially emerge under drug pressure.

Our analyses of species conservation and genetic diversity focused on the entire coding region rather than specific functional domains or regions involved in compound binding. It is conceivable that these domain regions exhibit higher conservation and may more accurately reflect the hypothesis that increased conservation correlates with greater essentiality. Additionally, gene vulnerability scores and essentiality predictions were determined under in vitro growth conditions, which may inaccurately classify certain genes as non-essential, such as those essential only during host infection. By examining published resistance-conferring mutations, we demonstrate the viability of some of these mutations within clinical populations, evidenced by the presence of several clinical isolates exhibiting inherent resistance. This underscores the considerable potential for these mutations, already established as viable in clinical settings, to be rapidly selected under drug pressure. However, our analysis is constrained by the availability of known resistance mutations, as it is highly probable that many resistance-conferring mutations remain unidentified, particularly for drug targets in early developmental stages. This highlights the urgent need for a comprehensive catalogue of resistance mutations prior to clinical deployment, which would serve as crucial resistance markers for future surveillance efforts. Furthermore, for several drugs in development, as well as those already in the clinic, the dominant mechanism of resistance are non-target based resistance mechanisms. For example, disruption of Rv0678 (MmpS5-MmpL5 efflux pump repressor) either by mutation, indel and/or frameshift leads to resistance against a broad spectrum of antibiotics including bedaquiline, clofazimine and Q203^37^. Finally, our dataset was assembled using available sequences mainly deposited in countries with low TB incidence but higher sequencing capacity. Whilst this is unavoidable, there is a potential that this distorts the true global distribution of clinical isolates. This is further compounded by the limited metadata attached to each isolate sequence, making it more challenging, for example, to identify ‘hotspots’ of potential resistance.

This comprehensive catalogue of genetic variance provides a baseline reference of *M. tuberculosis* genetic diversity on a single-gene level and can assist in future surveillance of drug resistance. Overall, the genetic baseline generated through our collated database and dashboard provides valuable information for understanding the genetic composition of *M. tuberculosis* populations in the clinic.

## Methods

The dataset was assembled from publicly available published sequence data (Supplementary Table S5). Sequence data in fastq format was downloaded from the European Nucleotide Archive (ENA). Reads were trimmed using Trimmomatic^38^ (v0.39; parameters: LEADING:3 TRAILING:3 SLIDINGWINDOW:4:20 MINLEN:36). Trimmed reads were aligned to the H37Rv reference genome (accession: NC000962.3) using bwa^39^ (v0.1.17). Duplicate aligned reads were marked with Samtools^40^ markdup (v1.12). Variants were then called using freebayes^41^ (v3.1.6) for each sample. For each sample, the predicted protein sequence was inferred by substituting in the variant calls into the reference and translating all genomic coding sequences into amino acid sequences. A custom script using biopython (v1.84; github.com/jodyphelan/genetic-diversity-db) was used to substitute variants into the reference genome. Low depth regions from each sample were inferred using Samtools depth and was used to mask the genomic sequence. Separately, TB-Profiler^42^ (v4.4.0; database version: e25540b) was used to predict drug resistance profile based on the presence of known drug resistance mutations and to assign a strain type. Linked data such as country of collection and date of collection were extracted from associated publications. To reduce the effect of oversampling from certain strain types, a subset of the data representative of the genetic diversity was generated by randomly selecting up to 10 different sequences from 117 different sublineages found by TB-Profiler. The percentage of polymorphic positions per gene was defined as the percentage of positions in a gene that have a polymorphism.

Species based conservation was calculated using protein sequences from *Mycobacterium tuberculosis* (ASM19595v2), *M. abscessus* (ASM6918v1), *M. avium* (ASM1498v1), *M. kansasii* (ASM15789v2), *M. marinum* (ASM1834v1) and *M. smegmatis* (ASM1500v1). Orthologues were found using OrthoFinder^43^ (v2.2.5) and orthogroups which had a single *M. tuberculosis* sequence were aligned using mafft^44^ (v7.520). Conservation by site was calculated at the percentage of alleles that were identical to the majority call. Gene conservation was calculated as the average of conservation across all sites. Association between the percent polymorphic positions across *M. tuberculosis*, species conservation and vulnerability scores^8^ was investigated by performing linear ordinary least squares regression using the regression.linear_model.OLS function from the statsmodels^45^ python package (v0.14.1). Figures were created using the plotly python package (v5.20.0). Sequence alignment was generated using ESPript 3.x software^46^.

Data preparation and ETL (extract, transform, and load) for aligned sequences was done using python version 3.9.7 to obtain the desired level of aggregation and output format for Tableau. The dashboard was developed in Tableau Desktop version 2023.3 and published to Tableau Public. The dashboard was subsequently integrated to the LSHTM website (https://www.lshtm.ac.uk/research/centres-projects-groups/satellite-centre-for-global-health-discovery#genetic-diversity) through an embedding code from Tableau Embedding API.

## Supplementary Information

Supplementary Information 1.

Supplementary Information 2.

## Data Availability

The datasets used in this study are publicly available via the European Nucleotide Archive (ENA) and the accession numbers are provided in Supplementary Table S5. Access to the dashboard generated in this project is available from the LSHTM website (https://www.lshtm.ac.uk/research/centres-projects-groups/satellite-centre-for-global-health-discovery#genetic-diversity).
