# Author Correction: An open-access dashboard to interrogate the genetic diversity of *Mycobacterium tuberculosis* clinical isolates

**DOI:** 10.1038/s41598-025-95856-4

**Published:** 2025-04-04

**Authors:** Jody Phelan, Klaas Van den Heede, Serge Masyn, Rudi Verbeeck, Taane G. Clark, Dirk A. Lamprecht, Anil Koul, Richard J. Wall

**Affiliations:** 1 Department of Infection Biology, Faculty of Infectious and Tropical Disease, London School of Hygiene and Tropical Medicine, London, WC1E 7HT UK; 2 Janssen Global Public Health R&D, LLC, Janssen Pharmaceutica NV, Turnhoutseweg 30, 2340 Beerse, Antwerpen, Belgium

Correction to: *Scientific Reports* 10.1038/s41598-024-75818-y, published online 21 October 2024

Taane G. Clark was omitted from the author list in the original version of this Article.

The Author Contributions section now reads:

“R.J.W., D.A.L. and A.K. conceived the project; J.P. compiled and analysed the clinical isolate dataset; T.G.C. provided datasets and analytic tools. K.V.H. assembled the dashboard interface; S.M. and R.V. supervised the dashboard assembly; R.J.W. wrote the manuscript with input from other authors. All authors reviewed the manuscript and approved the final version for publication.”

The original Article has been corrected.

