# Supplementary material for: An open-access dashboard to interrogate the genetic diversity of Mycobacterium tuberculosis clinical isolates
Source: Sci Rep. 2024 Oct 21;14:24792. doi: 10.1038/s41598-024-75818-y (PMC11494124; doi:10.1038/s41598-024-75818-y)
Supplement: Supplementary file 2 — Supplementary Information 2. [file 41598_2024_75818_MOESM2_ESM.docx]

SUPPLEMENTAL INFORMATION

**An open-access dashboard to interrogate the genetic diversity of *Mycobacterium tuberculosis* clinical isolates**

Jody Phelan^1^, Klaas Van den Heede^2,3^, Serge Masyn^2^, Rudi Verbeeck^2^, Dirk A. Lamprecht^2^, Anil Koul^1,2,*^ and Richard J. Wall^1,*^

^1^Department of Infection Biology, Faculty of Infectious and Tropical Disease, London School of Hygiene and Tropical Medicine, London, WC1E 7HT, UK

^2^Janssen Global Public Health R&D, LLC, Janssen Pharmaceutica NV, Turnhoutseweg 30, 2340 Beerse, Antwerpen, Belgium


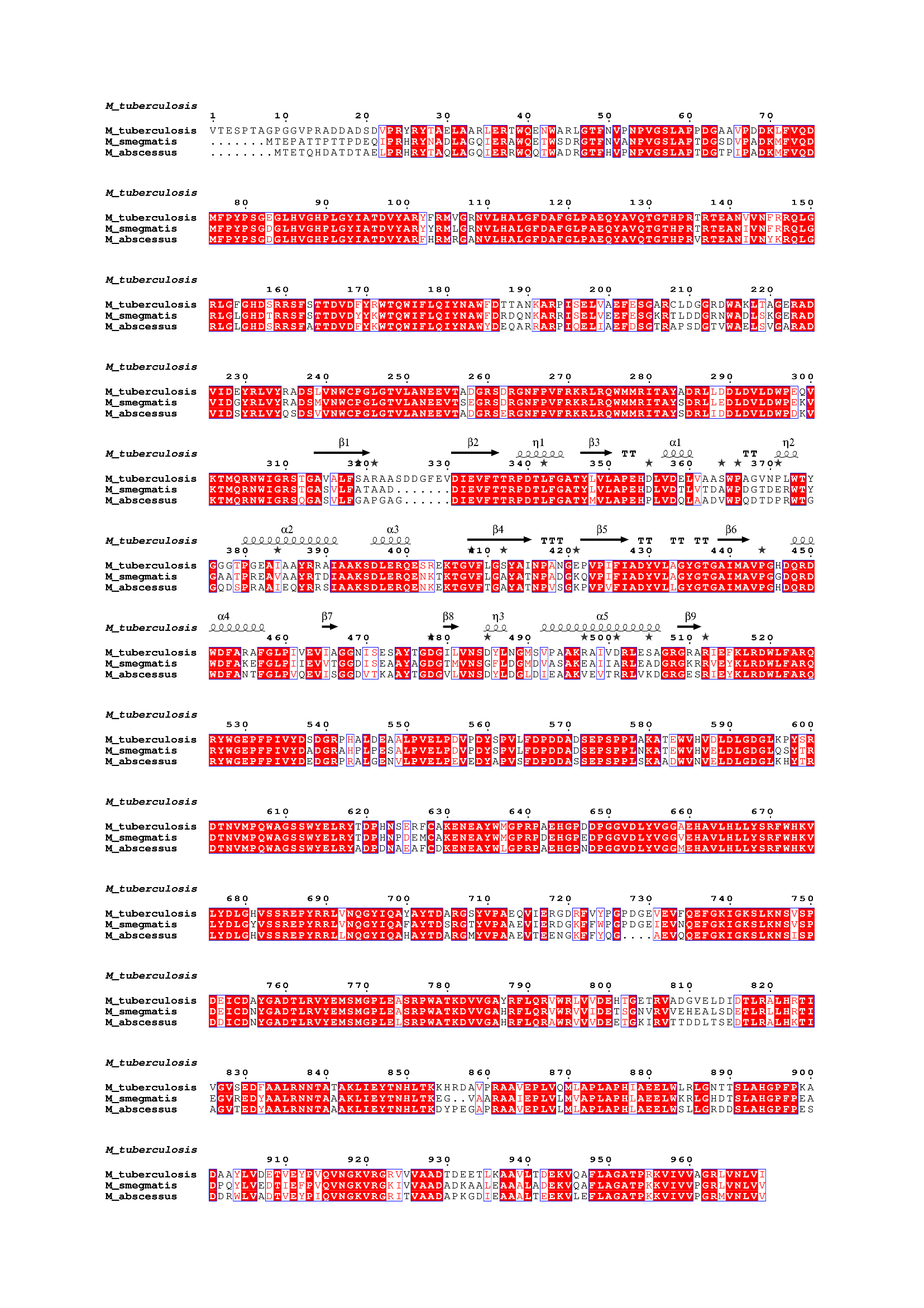


**Supplemental Figure S1: Alignment of LeuS in *Mycobacterium spp*.** – Protein structure was extrapolated from reference^1^ (PDB: 7PQK). Predicted compound binding site (within 5 angstroms distance of compound) is highlighted based on PDB model


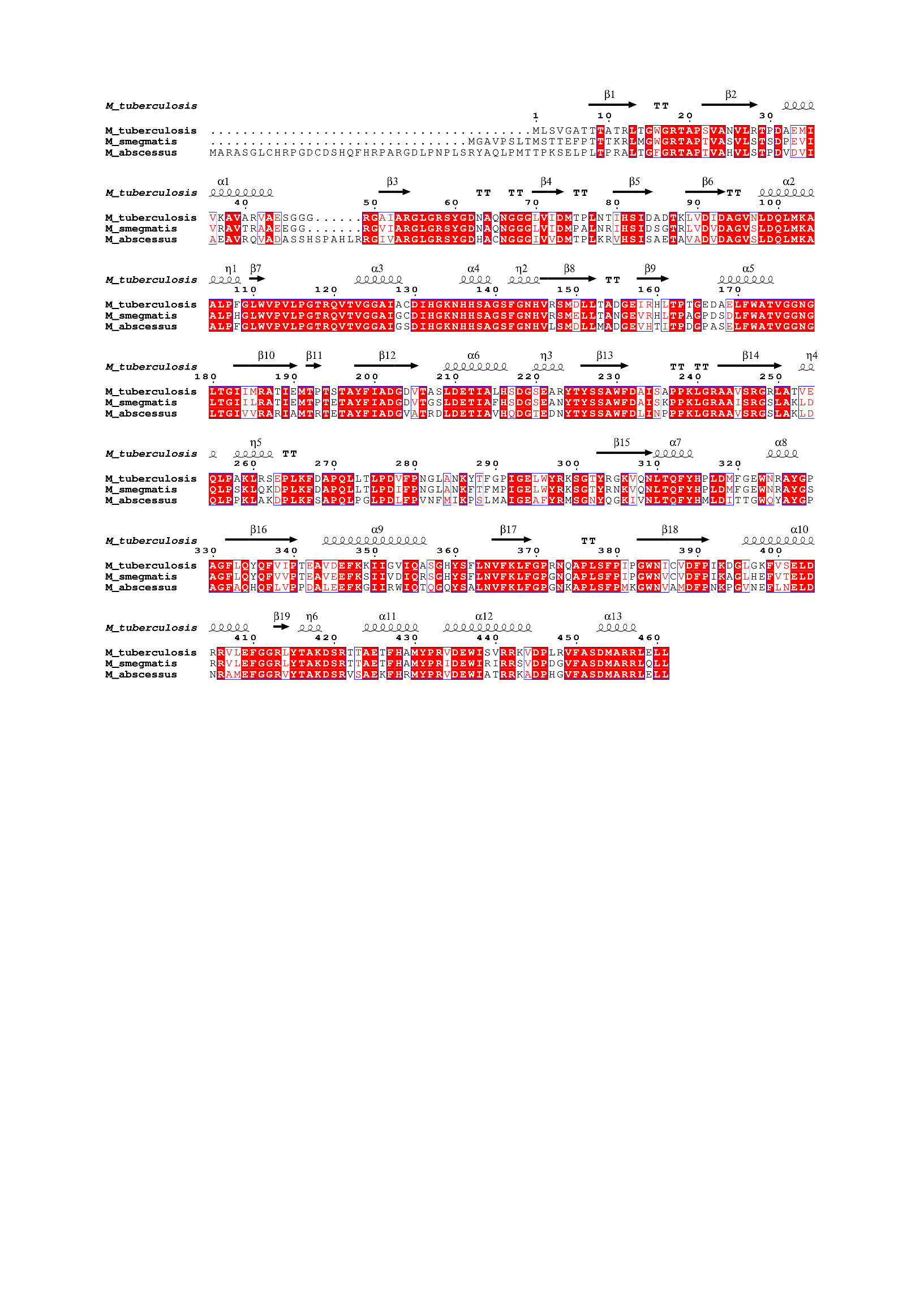


**Supplemental Figure S2: Alignment of DprE1 in *Mycobacterium spp.*** – Protein structure was extrapolated from reference^2^ (PDB: 4P8K). Predicted compound binding site (within 5 angstroms distance of compound) is highlighted based on PDB model.

**Supplemental Table S1: Comparison of vulnerability score, genetic diversity and species conservation**

**Supplemental Table S2: Genetic diversity and mutation analysis of Leucyl-tRNA Synthetase** – *M. tuberculosis* positions of mutations from other mycobacterium species was calculated based on sequence alignment (Supplemental Figure S1).

**Supplemental Table S3: Genetic diversity and mutation analysis of DprE1** – *M. tuberculosis* positions of mutations from other mycobacterium species was calculated based on sequence alignment (Supplemental Figure S2).

**Supplemental Table S4: Genetic diversity and mutation analysis of QcrB**

1. Hoffmann, G.; Le Gorrec, M.; Mestdach, E.; Cusack, S.; Salmon, L.; Jensen, M. R.; Palencia, A., Adenosine-Dependent Activation Mechanism of Prodrugs Targeting an Aminoacyl-tRNA Synthetase. *Journal of the American Chemical Society* **2023,** *145* (2), 800-810.

2. Neres, J.; Hartkoorn, R. C.; Chiarelli, L. R.; Gadupudi, R.; Pasca, M. R.; Mori, G.; Venturelli, A.; Savina, S.; Makarov, V.; Kolly, G. S.; Molteni, E.; Binda, C.; Dhar, N.; Ferrari, S.; Brodin, P.; Delorme, V.; Landry, V.; de Jesus Lopes Ribeiro, A. L.; Farina, D.; Saxena, P.; Pojer, F.; Carta, A.; Luciani, R.; Porta, A.; Zanoni, G.; De Rossi, E.; Costi, M. P.; Riccardi, G.; Cole, S. T., 2-Carboxyquinoxalines Kill Mycobacterium tuberculosis through Noncovalent Inhibition of DprE1. *ACS Chemical Biology* **2015,** *10* (3), 705-714.
